# The Distribution of Ocular Normative Parameters in a Spanish School Population

**DOI:** 10.3390/jcm14072507

**Published:** 2025-04-07

**Authors:** Rut González-Jiménez, F. Javier Povedano-Montero, Ricardo Bernárdez-Vilaboa, Rosario Gomez-de-Liano, Noemí Guemes-Villahoz, Juan E. Cedrún-Sánchez

**Affiliations:** 1 Optometry and Vision Department, Faculty of Optics and Optometry, Complutense University of Madrid, 28037 Madrid, Spain; rutgon03@ucm.es (R.G.-J.); ricardob@ucm.es (R.B.-V.); jcedrun@ucm.es (J.E.C.-S.); 2 Hospital Doce de Octubre Research Institute (i+12), 28041 Madrid, Spain; 3 San Carlos Clinical Hospital, 28040 Madrid, Spain; lianooft@ucm.es (R.G.-d.-L.); noemi.guemes@salud.madrid.org (N.G.-V.); 4 Instituto Investigaciones Oftalmologicas Ramos Castroviejo, Facultad de Medicina, Complutense University of Madrid, 28037 Madrid, Spain

**Keywords:** axial length, biometric parameters, corneal curvature, myopia, schoolchildren, Spain

## Abstract

**Background/Objectives**: The prevalence of myopia is increasing globally, including in Spain. The early detection of ocular biometric parameters associated with myopia development is crucial for implementing control strategies. This study aims to describe the normative biometric values in a Spanish school-aged population and compare them with previously established reference data. **Methods**: A cross-sectional, observational, and analytical study was conducted on 558 students aged 6 to 12 years from the Educare Valdefuentes School in Madrid. Ocular biometric parameters, including axial length (AL), corneal curvature (CR), anterior chamber depth (ACD), crystalline lens thickness (LT), corneal thickness (CCT), and posterior vitreous depth (PVD), were measured using IOLMaster 700. The axial length/corneal radius (AL/CR) ratio was calculated. Percentile growth curves were generated, and the results were statistically analyzed using IBM SPSS 29. **Results**: AL significantly increased with age (*p* < 0.001), and boys had longer AL than girls. The AL/CR ratio showed a moderate correlation with myopia risk (ρ = 0.647, *p* < 0.001). Compared to previous European studies, no significant differences were found, except for minor variations in AL and CR. **Conclusions**: These percentile-based biometric values provide a useful reference for monitoring ocular growth and assessing myopia risk in Spanish children. The AL/CR ratio remains a strong predictor of myopia development, supporting its role in early detection strategies.

## 1. Introduction

There is an increase in myopia in Spain, following the worldwide trend [1,2,3]. In a 2016 study, it was estimated that, by the year 2050, the myopic population will be 50% due to urbanization and modern lifestyles [4]. However, other research from 2024 predicts a myopia prevalence of 36.59% in 2040 and 39.80% in 2050 [5]. Sánchez Tena estimates that in Spain, myopia in children between 5 and 7 years of age will be 30.20% in 2050 [3]. In line with these forecasts, recent national data reveal that the prevalence of myopia in Spain was already 15.21% at age 5, 18.50% at age 6, and 21.90% at age 7 in 2021 [2,3], highlighting a clear upward trend from early childhood.

The increasing prevalence of myopia raises concerns due to its association with a higher risk of complications, including glaucoma, retinal detachment, and macular or choroidal neovascularization [6,7]. These complications can significantly impact health, finances, and socialization, ultimately reducing patients’ quality of life [8,9]. The early detection of children at high risk of developing myopia can help in its prevention and in implementing control strategies to stop its progression [10,11].

At birth, most infants’ eyes are hyperopic. They typically reach emmetropia around the age of six. Key ocular parameters, including axial length (AL), corneal curvature (CR), corneal thickness (CCT), and lens thickness (LT), influence eye growth. At birth, axial length (AL) ranges from 17 to 24 mm and increases to 21–26 mm in adulthood [12]. As for the cornea, its thickness does not vary much with age. The corneal curvature is more curved at birth and flattens by the age of 2 years [12]. The lens undergoes major changes with age; the thickness of the lens increases before birth and decreases until the age of 10–12 years, when it increases again [12,13]. The depth of the anterior chamber increases from birth until about 12 years of age, when it begins to decrease. The refractive error is the result of misalignments in these ocular parameters [12].

Although behavioral factors that may contribute to ocular dysregulation—such as limited outdoor exposure, intensive reading habits, or environmental factors—have been investigated, there is no robust evidence to confirm these associations [12,14,15]. Mutti et al. (2021) established that risk factors for developing myopia include parental myopia, time spent outdoors, and ethnicity; however, none of these factors were found to be predictive of myopia [10]. In contrast, when considering ocular parameters as risk factors or predictors of myopia, it is known that axial length (AL) and corneal radius (CR) are significant parameters for the assessment of myopia progression [16,17,18].

Myopic eyes exhibit a longer axial length (AL) compared to emmetropic or hyperopic eyes; consequently, an increase in the eye’s axial length is associated with an increase in myopia [19]. According to Mutti et al. (2007), ocular elongation begins approximately three years prior to the onset of myopia, with this elongation accelerating in the year immediately preceding its appearance [16]. Once myopia manifests, several factors must be considered, including an early age of onset, female sex, and ethnicity, all of which are linked to the more rapid progression of myopia [11,19,20,21,22]. Vera-Diaz et al. (2023) [23] report that the primary parameters predictive of myopia are AL and the AL/CR ratio (axial length to corneal radius ratio), with the latter offering greater precision as it accounts for both AL and corneal radius (CR). Accordingly, in children, the analysis of ocular parameters, particularly axial length and corneal curvature, may serve as a valuable tool for predicting future risk of developing myopia.

It is well established that AL varies across different racial groups and evolves over time. In Asian children (from Singapore), AL measurements were recorded as 23.90 ± 0.91 mm at age 7, 24.12 ± 0.89 mm at age 8, 24.52 ± 0.93 mm at age 9, and 25.05 ± 0.95 mm at age 11 [21]. In European children (from the Netherlands), the mean AL values were 22.36 ± 0.75 mm at age 6, 23.10 ± 0.84 mm at age 9, and 23.41 ± 0.86 mm at age 15 [17]. However, no reference data are available for pediatric populations in Spain.

The primary objective of this study is to characterize the parameters of ocular growth and to generate updated percentile curves for a Spanish population across different age groups. The obtained values will be analyzed and compared with currently available data [17] to determine whether trends suggest an increase in myopia at younger ages or, conversely, whether current AL values align with those previously established [8,12,17,21]. Additionally, this study aims to investigate the relationship between myopia and ocular parameters in Spanish children.

## 2. Materials and Methods

An observational, prospective, analytical, and cross-sectional study was conducted during the months of February and March 2024. A total of 558 students from Educare Valdefuentes School in Madrid, Spain, underwent ocular biometry measurements. The inclusion criterion required students to be enrolled in the primary education stage and to provide informed consent via forms signed by their parents or legal guardians. The exclusion criterion encompassed all students whose informed consent forms were not signed.

The students, predominantly Caucasian primary education pupils aged between 6 and 12 years, underwent ocular biometry measurements using an optical biometer, the IOLMaster700 Swept Source Biometry^®^ (Carl Zeiss Meditech AG, Jena, Germany).

### Measurement Procedure

Biometric measurements were obtained using the IOL Master 700 (Carl Zeiss Meditech), an optical coherence interferometry device that provides high precision in assessing ocular structures. Only measurements from the right eye were considered. The following parameters were measured:

- Axial length (AL);
- Corneal curvature (CR);
- Anterior chamber depth (ACD);
- Lens thickness (LT);
- Central corneal thickness (CCT);
- Posterior vitreous depth (PVD);
- Axial length/corneal curvature ratio (AL/CR).

Objective refraction was measured using retinoscopy at a distance to avoid stimulating accommodation, without the administration of cycloplegia. Myopia was defined as a spherical equivalent (SE), measured by retinoscopy without cycloplegia, of ≤−0.50 diopters (SE ≤ −0.50 D).

Statistical analysis was performed using IBM SPSS Statistics version 29.0.2.0. Using baseline cross-sectional data from the analyzed age groups (6, 7, 8, 9, 10, and 11 years), coefficients were calculated for AL, CR, AL/CR ratio, ACD, CCT, and PVD. Cutoff points were determined for the 5th, 10th, 25th, 50th, 75th, 90th, and 95th percentiles. These percentiles were selected to enable comparison with existing percentile tables for European children. Correlations were calculated between variables. The data obtained were compared with findings from previously published studies. Sensitivity and specificity were assessed to evaluate the utility of cross-sectional percentile cutoffs in predicting the development of myopia among students. Receiver operating characteristic (ROC) curve analysis was conducted; for this calculation, the total number of eyes was reduced to 508 due to SE data being unavailable.

This study was approved by the Research Ethics Committee for Medicinal Products of the San Carlos Clinical Hospital (23/425-E) and was conducted in accordance with the Declaration of Helsinki.

## 3. Results

### 3.1. Demographic and Ocular Characteristics

The study included a total of 558 school-aged children, comprising 259 males (46%) and 299 females (54%), with ages ranging from 5 to 12 years (mean ± SD: 8.50 ± 1.72 years). Demographic and ocular biometric characteristics are summarized in Table 1 and Table 2. Table 1 shows a statistically significant difference in AL/CR ratio between sexes (*p* = 0.03), although this value corresponds to the overall comparison without considering age stratification. When analyzed by age group (Table 2), a significant sex-related difference in AL/CR was only observed in the 11-year-old group (*p* = 0.009), whereas differences were not significant in the remaining age groups.

**Figure 1:**
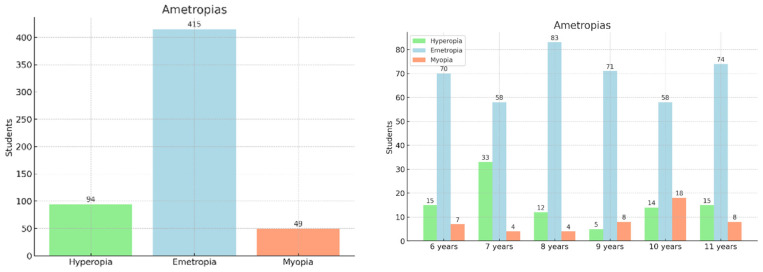
The distribution of refractive errors in the total sample and by age group.

The overall prevalence of refractive errors in this study was 8.78% for myopia, 16.85% for hyperopia, and 74.37% for emmetropia. Figure 1 illustrates the distribution of refractive status in the total sample and across different age groups, respectively. These visualizations highlight the progressive increase in the proportion of myopic children as age advances, alongside a decline in hyperopia. No statistically significant differences were found in refractive error distribution between sexes.

### 3.2. Growth Curves and Percentiles of Ocular Parameters

**Figure 2:**
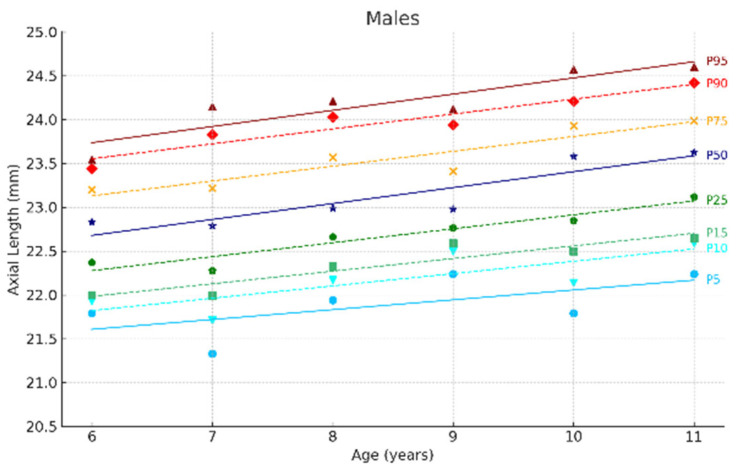
Axial length growth curves of the eye for males aged 6 to 11 years. Overall, 5 to 95 represent the 5th to 95th percentiles. Males N = 259 (6 years = 45, 7 years = 45, 8 years = 48, 9 years = 40, 10 years = 41, 11 years = 39).

**Figure 3:**
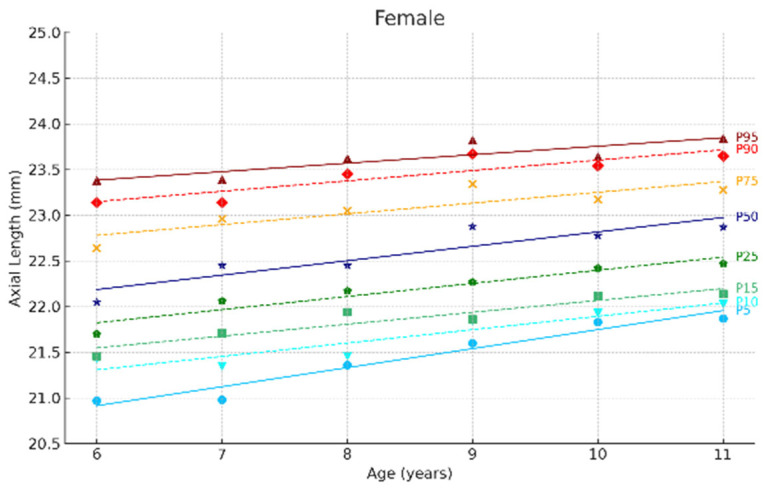
Axial length growth curves of the eye for females aged 6 to 11 years. Overall, 5 to 95 represent the 5th to 95th percentiles. Females N = 299 (6 years = 47, 7 years = 50, 8 years = 51, 9 years = 44, 10 years = 49, 11 years = 58).

**Figure 4:**
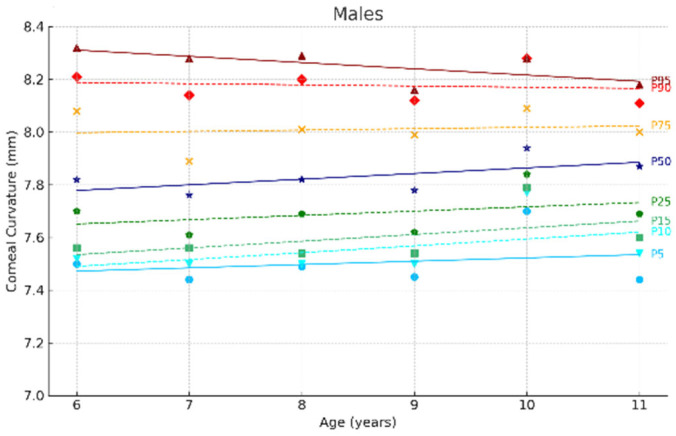
Corneal curvature growth curves of the eye for males aged 6 to 11 years. Overall, 5 to 95 represent the 5th to 95th percentiles. Males N = 259 (6 years = 45, 7 years = 45, 8 years = 48, 9 years = 40, 10 years = 41, 11 years = 39).

**Figure 5:**
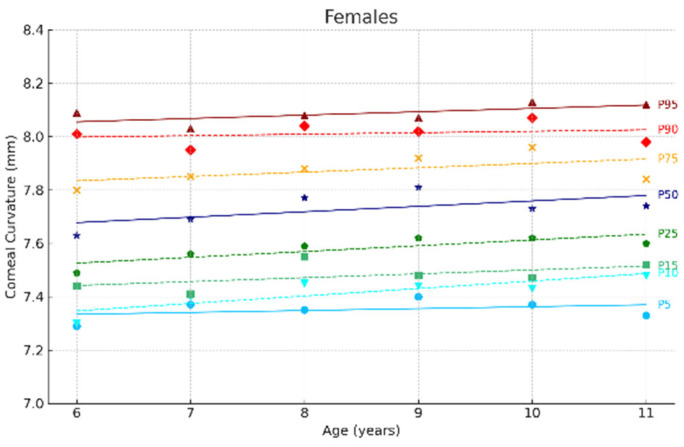
Corneal curvature growth curves of the eye for females aged 6 to 11 years. Overall, 5 to 95 represent the 5th to 95th percentiles. Females N = 299 (6 years = 47, 7 years = 50, 8 years = 51, 9 years = 44, 10 years = 49, 11 years = 58).

**Figure 6:**
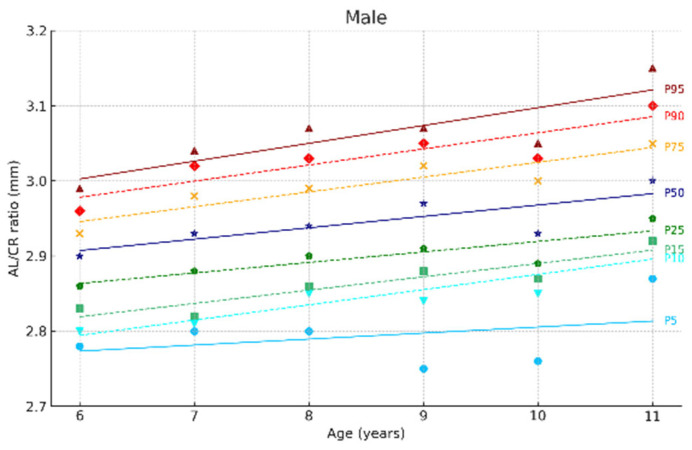
Al/CR ratio growth curves of the eye for males aged 6 to 11 Years. Overall, 5 to 95 represent the 5th to 95th percentiles. Males N = 259 (6 years = 45, 7 years = 45, 8 years = 48, 9 years = 40, 10 years = 41, 11 years = 39).

**Figure 7:**
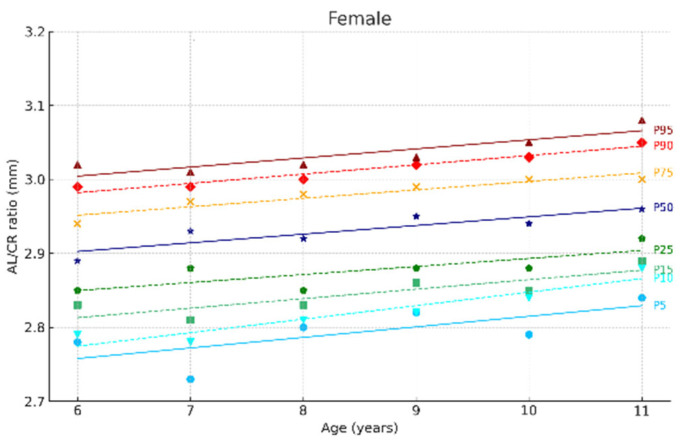
Al/CR ratio growth curves of the eye for females aged 6 to 11 Years. Overall, 5 to 95 represent the 5th to 95th percentiles. Females N = 299 (6 years = 47, 7 years = 50, 8 years = 51, 9 years = 44, 10 years = 49, 11 years = 58).

Figure 2 and Figure 3 show the ocular growth curves for males and females across each age group for axial length (AL), corneal radius (CR), and the AL/CR ratio. Growth curves for anterior chamber depth (ACD), posterior vitreous distance (PVD), lens thickness (LT), and central corneal thickness (CCT) are presented in Figure 4 and Figure 5 and in the Supplementary Material. Figure 6 and Figure 7 represent the growth curves of the axial length to corneal radius ratio (AL/CR) for males and females. The corresponding percentile values are provided in Table S1 (Supplementary Material). Overall, for both sexes among the analyzed age groups, the growth curves exhibited a linear trend. A statistically significant increase was observed in AL with age in both genders across all percentiles (*p* < 0.001). A similar trend was found for the other ocular biometric parameters.

### 3.3. Correlations Between Ocular Parameters

The strongest correlation obtained for the parameters studied was between ACD RE and PVD RE, with ρ = 0.992 and *p* < 0.001. Moderate correlations were observed for AL and CR (ρ = 0.642, *p* < 0.001), AL/CR and ACD (ρ = 0.647, *p* < 0.001), and the AL/CR ratio and PVD (ρ = 0.653, *p* < 0.001). The correlations obtained are presented in Table 3. These correlations remained similar when the sample was filtered by age.

### 3.4. Comparison of Spanish vs. European Parameters

When comparing the values obtained in this study with those reported for Dutch patients, no significant differences were found in the median values for ages 6 and 9 years, except for the AL in males at 6 years, which was greater and statistically significant in this study, and the CR in females at 9 years, which was more curved and significantly different in our study. These data are presented in Table 4. Table S2a,b (Supplementary Material) display the values obtained for the studied percentiles and their comparison with Dutch eye values for ages 6 and 9 years. For the age of 6 years, AL was significantly higher in females in this study at the 10th percentile, and the AL/CR ratio in females was lower at the 95th percentile compared to the values previously reported for Dutch girls. For the age of 9 years, in males, AL values were longer in this study at the 90th and 95th percentiles compared to Dutch patients; in females, the 25th and 50th percentiles showed more curved corneal curvatures, and at the 90th and 95th percentiles, the AL/CR ratio was higher in Spanish girls than in Dutch girls.

### 3.5. ROC Curve Analysis for Myopia Prediction Using Ocular Parameters

**Figure 8:**
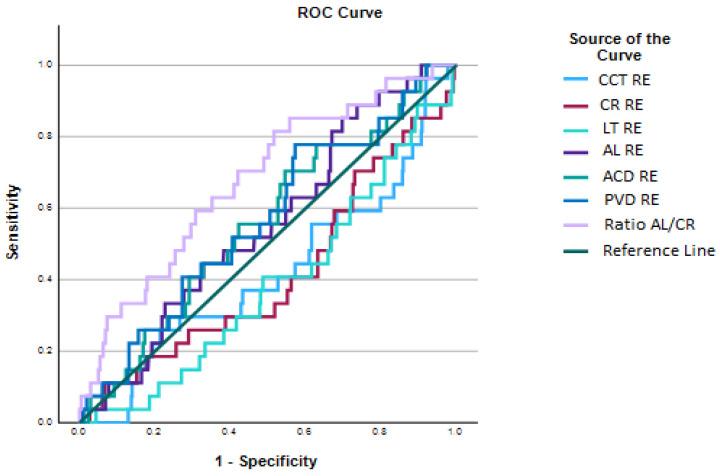
Receiver operating characteristic (ROC) curves for the ocular parameters analyzed.

Figure 8 presents the ROC curve analysis. Sensitivity indicates the proportion of true positives, while specificity reflects the proportion of false positives. Each curve evaluates how effectively each ocular parameter distinguishes between children with and without myopia. The diagonal reference line represents random performance (AUC = 0.5), whereas curves closer to the upper left corner indicate better predictive performance. The best individual predictor of myopia was the AL/CR ratio, which showed the curve furthest from the reference line (AUC = 0.679, *p* = 0.001, 95% CI: 0.576–0.782), suggesting moderate discriminatory ability for myopia. Other variables, such as PVD (AUC = 0.566, *p* = 0.235) and ACD (AUC = 0.559, *p* = 0.286), exhibited low discriminatory capacity. However, LT (AUC = 0.388, *p* = 0.035) showed a classification performance below random. The AUC values for each parameter, along with their 95% confidence intervals, are detailed in Table 5.

**Figure 9:**
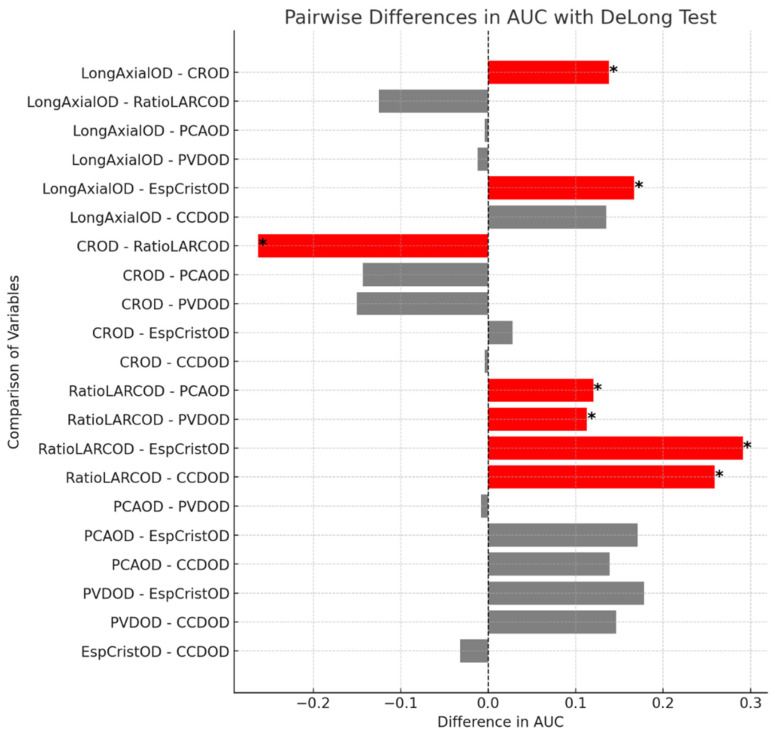
Pairwise comparisons of AUC differences between ocular biometric parameters using DeLong test. Red bars indicate statistically significant differences (*p* < 0.05), gray bars indicate non-significant differences (*p* ≥ 0.05), and an asterisk (*) highlights the comparisons with statistically significant results.

The results of the DeLong test comparing the area under the ROC curves (AUCs) are presented in Figure 9. This analysis highlights that the AL/CR ratio exhibits significantly greater discriminative power for detecting myopia when compared to several other biometric variables. Each bar in the figure represents the difference in AUC between two variables: red bars indicate statistically significant differences (*p* < 0.05), gray bars indicate non-significant differences (*p* ≥ 0.05), and an asterisk (*) highlights comparisons with significant differences. These findings reinforce the relevance of AL/CR as a robust predictor in early myopia detection strategies.

## 4. Discussion

The results of this study provide updated normative values for ocular biometry in Spanish schoolchildren, enhancing the understanding of ocular growth in this population and its relationship with myopia development.

This study evaluated ocular parameters and their progression toward emmetropization in a population of Spanish students, and results aligned with the expected growth patterns estimated by other authors [12,13,24,25]. AL increased significantly between the ages of 6 and 11 years, with greater values in males than in females. CCT showed little variation with age [12]. LT decreased up to 12 years, although data were only available up to 11 years, consistent with reductions observed in the studied age range [24,26]. ACD increased up to 12 years, in agreement with the data obtained in this study.

Early indicators of future myopia may appear several years before its clinical onset, yet excessive axial elongation can remain undetected due to compensatory reductions in the lens’s dioptric power [12]. In this study, weak correlation was found between axial length (AL) and lens thickness (LT), possibly due to the absence of cycloplegia—which may affect lens measurements—or due to lens positioning. In contrast, the strong correlation between anterior chamber depth (ACD) and posterior vitreous depth (PVD) suggests that axial elongation is not uniform, which may be critical in identifying the type and progression pattern of myopia in each patient.

Based on the values obtained and the previously published literature, an AL exceeding the expected percentiles for a given age is associated with an increased risk of developing myopia. As percentiles rise above the 50th, the likelihood of myopia increases [17]. An AL/CR ratio ≥ 3.00 has also been shown to correlate with a higher risk of myopia [8,12]. Additionally, according to several authors (Mutti, Fuensanta, Zadnik, and Jiong [16,23,27,28]), children with thicker lenses are at greater risk of developing myopia. These percentiles can serve as valuable tools for detecting abnormal eye growth, like normative values for weight or height in children.

Given the various studies reporting increases in myopia [1,2,3,4,5], questions arose about whether growth curves used as references in ophthalmology, based on a Dutch population [17], remain appropriate. Due to the lack of complete data from that study [17], we calculated the median and confidence interval (CI) to determine if Tideman’s means fell within our study’s CI, thus assessing whether differences were statistically significant. Differences were minimal: AL in males at 6 years was slightly higher, and CR in females at 9 years was slightly more curved. For AL, this may be attributed to our sample comprising Spanish males compared to the Dutch males in featured Tideman’s study. For CR, the difference was only 0.05 mm (lower CI limit), which is not clinically significant. For percentile comparisons, confidence intervals were calculated via sampling simulation.

The ROC curve analysis indicates that, among the parameters studied, the AL/CR ratio is the strongest predictor of myopia, which is consistent with previous studies [12,23,29,30,31,32,33,34] suggesting that AL/CR is the most accurate biometric predictor of myopia, although not yet precise enough to serve as a standalone diagnostic tool [23]. In the present study, this finding was further supported by pairwise comparisons of AUC values using DeLong’s test (Figure 9), which demonstrated statistically significant differences between AL/CR and several other biometric variables. These results reinforce the discriminative power of the AL/CR ratio and its value in early myopia detection strategies.

The strength of this study lies in the sample size and comprehensive description of ocular parameters. However, several limitations warrant discussion. The primary limitation is the inability to perform pupil dilation in the students. Although refraction was measured at a distance, uncontrolled accommodation studied may introduce bias. Another limitation is that, as noted in the results, refraction data were unavailable or imprecise for 50 students, causing them to be excluded from the ROC curve analysis.

Additionally, it is relevant to investigate whether significant differences exist in biometric parameter progression across different regions of Spain, considering potential variations in environmental factors and visual habits. A more detailed analysis of screen time exposure and outdoor activity could establish more precise correlations with myopia progression in this population.

Another consideration is the need for longitudinal follow-up to confirm the evolution of these biometric parameters and their impact on myopia progression over time. Future research could also explore the influence of environmental factors on ocular development and assess the efficacy of specific interventions for myopia control.

This study suggests future investigations, such as re-evaluating the sample in three years to assess changes and exploring the feasibility of initiating controls before the onset of myopia, particularly in patients identified as high-risk based on ocular parameters.

## 5. Conclusions

The values obtained for the growth of ocular biometric parameters in Spanish children are consistent with those previously described in the scientific literature. The evolution of axial length and other parameters related to ocular development follows patterns similar to those reported in prior studies.

Percentiles generated from the 2024 Spanish school population show high concordance with those obtained 15 years ago, which remain in use as references today. This suggests stability in normative ocular growth values, supporting their utility as comparative tools in pediatric ophthalmologic assessments.

The obtained percentiles may serve as useful tools for the early detection of abnormal eye growth, akin to the use of normative values for weight and height in pediatrics. Their application in clinical practice could facilitate the early identification of children at risk of progressive myopia or ocular anomalies.

Among the analyzed parameters, the axial length/corneal curvature ratio (AL/CR) was identified as the best predictor of myopia development in Spanish children. This finding reinforces its value in screening and monitoring strategies for pediatric visual health, enabling the implementation of tailored preventive and therapeutic measures.

## Figures and Tables

**Table 1:** Demographic and ocular characteristics of the students’ participants of this study. Median (IQR) (min, max).

	Male	Female	*p* Value
	Median (IQR) Min–Max	Median (IQR) Min–Max	
No. of eyes (n)	259	299	
Age (years)	8.00 (7.00–10.00) (6.00, 12.00)	9.00 (7.00–10.00) (6.00, 12.00)	
Axial length (mm) (AL)	23.04 (22.60–23.64) (20.34, 25.59)	22.59 (22.12–23.12) (19.64, 25.23)	*p* < 0.001
Corneal curvature (mm) (CR)	7.83 (7.68–8.02) (7.18, 8.79)	7.74 (7.56–7.87) (7.00, 8.48)	*p* < 0.001
AL/CR	2.94 (2.90–3.00) (2.65, 3.25)	2.93 (2.88–2.98) (2.63, 3.19)	*p* = 0.03
Anterior chamber depth (mm) (ACD)	3.59 (3.42–3.75) (2.90, 4.30)	3.51 (3.31–3.65) (2.72, 4.07)	*p* < 0.001
Posterior vitreous depth (mm) (PVD)	3.04 (2.86–3.19) (2.31, 3.70)	2.95 (2.77–3.10) (2.19, 3.57)	*p* < 0.001
Crystalline lens thickness (mm) (LT)	3.47 (3.33–3.60) (3.05, 4.05)	3.52 (3.40–3.63) (3.01, 4.16)	*p* = 0.003
Corneal thickness (mm) (CCT)	0.56 (0.54–0.58) (0.47, 0.67)	0.55 (0.53–0.57) (0.48, 0.64)	*p* < 0.001

**Table 2:** Demographic and ocular characteristics for the student’s age groups. Median (IQR) (min, max).

		6 Years	7 Years	8 Years	9 Years	10 Years	11 Years	*p* Value
No. of eyes (n)	Male	45	45	48	40	41	39	
	Female	47	50	51	44	49	58	
Axial length (mm) (AL)	Male	22.83 (22.36–23.22) (21.22, 24.19)	22.79 (22.18–22.23) (20.34, 24.61)	22.99 (22.66–23.58) (21.37, 25.34)	22.98 (22.75–23.44) (20.77, 24.32)	23.58 (22.83–23.94) (21.43, 25.41)	23.63 (23.12–23.99) (21.76, 25.59)	*p* < 0.001
	Female	22.05 (21.70–22.64) (20.32, 23.89)	22.45 (22.06–22.97) (16.64, 23.60)	22.45 (22.17–23.05) (20.70, 23.88)	22.88 (22.27–23.35) (20.83, 24.73)	22.78 (22.40–23.20) (21.26, 23.78)	22.87 (22.46–23.28) (21.48, 25.23)	
Corneal curvature (mm) (CR)	Male	7.82 (7.69–8.09) (7.37, 8.63)	7.76 (7.60–7.92) (7.18, 8.42)	7.82 (7.68–8.01) (7.26, 8.48)	7.78 (7.61–7.99) (7.41, 8.39)	7.94 (7.83–8.09) (7.20, 8.79)	7.87 (7.69–8.00) (7.33, 8.43)	*p* = 0.013
	Female	7.63 (7.49–7.80) (7.00, 8.30)	7.69 (7.54–7.85) (7.17, 8.13)	7.77 (7.58–7.88 (7.09, 8.31)	7.81 (7.30–7.92) (7.25, 8.48)	7.73 (7.60–7.97) (7.17, 8.35)	7.74 (7.59–7.84) (7.28, 8.33)	
AL/CR	Male	2.90 (2.85–2.94) (2.66, 3.06)	2.93 (2.88–2.98) (2.68, 3.09)	2.94 (2.90–2.99) (2.75, 3.16)	2.97 (2.91–3.02) (2.69, 3.15)	2.94 (2.89–3.00) (2.65, 3.11)	3.00 (2.95–3.05) (2.87, 3.25)	*p* < 0.001
	Female	2.89 (2.85–2.94) (2.73, 3.05)	2.93 (2.88–2.97) (2.67, 3.08)	2.92 (2.85–2.98) (2.64, 3.03)	2.95 (2.88–2.99) (2.75, 3.06)	2.94 (2.88–3.00) (2.70, 3.19)	2.96 (2.92–3.00) (2.63, 3.14)	
Anterior chamber depth (mm) (ACD)	Male	3.51 (3.38–3.63) (3.01.3.95)	3.55 (3.39–3.69) (2.94, 3.92)	3.61 (3.42–3.74) (3.10, 4.23)	3.56 (3.40–3.76) (3.11, 4.11)	3.65 (3.40–3.85) (2.90, 4.11)	3.78 (3.59–3.93) (3.18, 4.30)	*p* < 0.001
	Female	3.38 (3.29–3.58) (2.94, 4.04)	3.58 (3.28–3.73) (2.93, 4.06)	3.48 (3.24–3.68) (2.72, 3.98)	3.48 (3.38–3.67) (3.09, 4.07)	3.53 (3.36–3.68) (2.92, 4.04)	3.54 (3.37–3.65) (2.84, 4.02)	
Posterior vitreous depth(mm) (PVD)	Male	2.96 (2.81–3.06) (2.50, 3.38)	3.01 (2.86–3.12) (2.38, 3.34)	3.06 (2.86–3.19) (2.51, 3.65)	2.98 (2.83–3.19) (2.60, 3.54)	3.08 (2.84–3.26) (2.31, 3.53)	3.20 (3.02–3.34) (2.57, 3.71)	*p* = 0.001
	Female	2.83 (2.76–3.03) (2.36, 3.50)	3.01 (2.72–3.17) (2.43, 3.55)	2.90 (2.70–3.10) (2.19, 3.49)	2.92 (2.81–3.07) (2.54, 3.57)	2.97 (2.82–3.13) (2.37, 3.49)	3.00 (2.84–3.11) (2.32, 3.47)	
Crystalline lens thickness (mm) (LT)	Male	3.60 (3.39–3.71) (3.15, 4.02)	3.47 (3.35–3.59) (3.18, 4.05)	3.47 (3.32–3.61) (3.12, 3.83)	3.49 (3.37–3.59) (3.05, 3.86)	3.46 (3.31–3.52) (3.15, 3.88)	3.40 (3.29–3.51) (3.05, 3.96)	*p* < 0.001
	Female	3.59 (3.49–3.67) (3.30, 4.04)	3.55 (3.40–3.67) (3.15, 3.94)	3.52 (3.39–3.68) (3.25, 4.16)	3.47 (3.36–3.60) (3.14, 3.96)	3.48 (3.40–3.55) (3.01, 3.96)	3.50 (3.41–3.58) (3.06, 3.91)	
Corneal thickness (mm) (CCT)	Male	0.56 (0.54–0.58) (0.48, 0.63)	0.55 (0.53–0.57) (0.47, 0.63)	0.56 (0.54–0.58) (0.50, 0.67)	0.56 (0.54–0.59) (0.49, 0.66)	0.56 (0.52–0.58) (0.48, 0.63)	0.56 (0.54–0.59) (0.48, 0.63)	*p* = 0.421
	Female	0.55 (0.53–0.56) (0.50, 0.60)	0.55 (0.52–0.57) (0.48, 0.59)	0.55 (0.53–0.57) (0.48, 0.63)	0.55 (0.52–0.57) (0.48, 00.64)	0.55 (0.52–057) (0.48, 0.62)	0.55 (0.53–0.56) (0.50, 0.62)	

**Table 3:** Spearman’s correlations.

	AL	CR	RAL/CR	ACD	PVD	LT	CCT
AL	1	0.642 **	0.443 **	0.483**	0.471**	−0.373 **	0.150 **
CR	0.642 **	1	−0.331 **	0.003	−0.017	0.023	0.168 **
AL/CR	0.443 **	−0.331 **	1	0.647 **	0.653 **	−0.531 **	−0.002
ACD	0.483 **	0.003	0.647 **	1	0.992 **	−0.475 **	0.104 *
PVD	0.471 **	−0.017	0.653 **	0.992 **	1	−0.480 **	−0.008
LT	−0.373 **	0.023	−0.531 **	−0.475 **	−0.480 **	1	0.001
CCT RE	0.150 **	0.168 **	−0.002	0.104 *	−0.008	0.001	1

** correlation is significant at the 0.01 level (2-tailed). * correlation is significant at the 0.05 level (2-tailed).

**Table 4:** A comparison of the means and confidence intervals of the data obtained with the median values reported in the Tideman 2018 study [17].

				Valid N	Median	95.0% Lower CI for Median	95.0% Upper CI for Median	Tideman, 2018 [17]	Statistical Significance
AL	Age	6	Female	47	22.05	21.9	22.34	22.06	NO
			Male	45	22.83	22.56	23.02	22.59	NO
		9	Female	44	22.88	22.58	23.17	22.79	NO
			Male	40	22.98	22.85	23.21	23.31	SI
CR	Age	6	Female	47	7.63	7.55	7.75	7.7	NO
			Male	45	7.82	7.77	7.94	7.84	NO
		9	Female	44	7.81	7.77	7.89	7.72	SI
			Male	40	7.78	7.72	7.91	7.84	NO
LA/RC	Age	6	Female	47	2.89	2.87	2.92	2.87	NO
			Male	45	2.9	2.87	2.92	2.89	NO
		9	Female	44	2.95	2.9	2.97	2.95	NO
			Male	40	2.97	2.94	3	2.97	NO

**Table 5:** AUC values for each ocular parameter. ^a^ Standard Error under the nonparametric assumption. ^b^ Null hypothesis: true area = 0.5.

Area Under the ROC Curve					
Test Result Variable(s)	Area	Std. Error ^a^	Asymptotic Sig. ^b^	Asymptotic 95% Confidence Interval	
				Lower Bound	Upper Bound
AL	0.555	0.052	0.294	0.453	0.656
CR	0.416	0.059	0.155	0.301	0.532
LA/CR	0.679	0.052	0.001	0.576	0.782
ACD	0.559	0.055	0.286	0.451	0.666
PVD	0.566	0.056	0.235	0.457	0.675
LT	0.388	0.053	0.035	0.284	0.492
CCT	0.42	0.059	0.178	0.304	0.536

## Data Availability

The data supporting the conclusions of this study are available upon request from the corresponding author. Due to ethical and privacy restrictions, the data are not publicly accessible.

## Supplementary Materials

The following supporting information can be downloaded at: https://www.mdpi.com/article/10.3390/jcm14072507/s1, Table S1. Corneal cut-off parameters for the different percentiles with upper and lower 95% confidence intervals for males and females of the reference ages. Tabla S2: (a) 6 year: Comparison this study vs. Tideman, 2018 [17]. Sampling simulation. (b) 9 year: Comparison this study vs. Tideman, 2018 [17]. Sampling simulation. Figure S1. ACD growth curves of the eye for Males aged 6 to 11 Years. 5 to 95 represent the 5th to 95th percentiles. Males N = 259 (6 years = 45, 7 years = 45, 8 years = 48, 9 years = 40, 10 years = 41, 11 years = 39). Figure S2. ACD growth curves of the eye for Females aged 6 to 11 Years. 5 to 95 represent the 5th to 95th percentiles. Females N = 299 (6 years = 47, 7 years = 50, 8 years = 51, 9 years = 44, 10 years = 49, 11 years = 58). Figure S3. PVD growth curves of the eye for Males aged 6 to 11 Years. 5 to 95 represent the 5th to 95th percentiles. Males N = 259 (6 years = 45, 7 years = 45, 8 years = 48, 9 years = 40, 10 years = 41, 11 years = 39). Figure S4. PVD growth curves of the eye for Females aged 6 to 11 Years. 5 to 95 represent the 5th to 95th percentiles. Females N = 299 (6 years = 47, 7 years = 50, 8 years = 51, 9 years = 44, 10 years = 49, 11 years = 58). Figure S5. LT growth curves of the eye for Males aged 6 to 11 Years. 5 to 95 represent the 5th to 95th percentiles. Males N = 259 (6 years = 45, 7 years = 45, 8 years = 48, 9 years = 40, 10 years = 41, 11 years = 39). Figure S6. LT growth curves of the eye for Females aged 6 to 11 Years. 5 to 95 represent the 5th to 95th percentiles. Females N = 299 (6 years = 47, 7 years = 50, 8 years = 51, 9 years = 44, 10 years = 49, 11 years = 58). Figure S7. CCT growth curves of the eye for Males aged 6 to 11 Years. 5 to 95 represent the 5th to 95th percentiles. Males N = 259 (6 years = 45, 7 years = 45, 8 years = 48, 9 years = 40, 10 years = 41, 11 years = 39). Figure S8. CCT growth curves of the eye for Males and Females aged 6 to 11 Years. 5 to 95 represent the 5th to 95th percentiles. Females N = 299 (6 years = 47, 7 years = 50, 8 years = 51, 9 years = 44, 10 years = 49, 11 years = 58).

## Abbreviations

The following abbreviations are used in this manuscript:

AL	Axial length
CR	Corneal curvature
AL/CR	AL/CR ratio
ACD	Anterior chamber depth
PVD	Posterior vitreous depth
LT	Lens thickness
CCT	Central corneal thickness
SE	spherical equivalent
