# Supplementary material for: The Distribution of Ocular Normative Parameters in a Spanish School Population
Source: J Clin Med. 2025 Apr 7;14(7):2507. doi: 10.3390/jcm14072507 (PMC11989478; doi:10.3390/jcm14072507)
Supplement: Supplementary file 1 [file jcm-14-02507-s001.zip › jcm-3538775-supplementary.pdf]

Supplementary Material:

Table S1. Corneal cut-off parameters for the different percentiles with upper and lower 95% confidence intervals for males and females of the reference ages.

Age			N	P5	P10	P15	P25	P50	P75	P90	P95
**6 years**	**AL**	**Female**	47	21	21,43	21,46	21,7	22,05	22,64	23,14	23,38
		**Male**	45	21,8	21,93	22	22,4	22,83	23,2	23,44	23,54
		**Total**	92	21,4	21,47	21,71	21,9	22,44	22,97	23,38	23,54
	**CR**	**Female**	47	7,29	7,3	7,44	7,49	7,63	7,8	8,01	8,09
		**Male**	45	7,5	7,52	7,56	7,7	7,82	8,08	8,21	8,32
		**Total**	92	7,3	7,44	7,49	7,56	7,75	7,92	8,15	8,22
	**AL/RC**	**Female**	47	2,78	2,79	2,83	2,85	2,89	2,94	2,99	3,02
		**Male**	45	2,78	2,8	2,83	2,86	2,9	2,93	2,96	2,99
		**Total**	92	2,78	2,8	2,83	2,85	2,9	2,94	2,98	3,02
	**ACD**	**Female**	47	2,99	3,14	3,18	3,29	3,38	3,58	3,66	3,76
		**Male**	45	3,12	3,18	3,21	3,4	3,51	3,6	3,75	3,87
		**Total**	92	3,06	3,15	3,21	3,3	3,47	3,59	3,73	3,79
	**PVD**	**Female**	47	2,45	2,6	2,64	2,76	2,83	3,03	3,12	3,21
		**Male**	45	2,55	2,62	2,65	2,82	2,96	3,06	3,21	3,35
		**Total**	92	2,53	2,62	2,64	2,76	2,9	3,04	3,16	3,26
	**LT**	**Female**	47	3,38	3,4	3,45	3,49	3,59	3,67	3,74	3,92
		**Male**	45	3,25	3,3	3,35	3,4	3,6	3,71	3,81	3,88
		**Total**	92	3,3	3,36	3,38	3,45	3,59	3,7	3,76	3,9
	**CCR**	**Female**	47	0,51	0,52	0,53	0,53	0,55	0,56	0,58	0,59
		**Male**	45	0,51	0,51	0,52	0,54	0,56	0,58	0,59	0,61
		**Total**	92	0,51	0,52	0,52	0,53	0,55	0,57	0,59	0,6
**7 years**	**AL**	**Female**	50	21	21,35	21,71	22,1	22,45	22,96	23,14	23,39
		**Male**	45	21,3	21,71	21,99	22,3	22,79	23,22	23,83	24,15
		**Total**	95	21,1	21,64	21,83	22,1	22,64	23,06	23,43	23,83
	**CR**	**Female**	50	7,37	7,4	7,41	7,56	7,69	7,85	7,95	8,03
		**Male**	45	7,44	7,5	7,56	7,61	7,76	7,89	8,14	8,28
		**Total**	95	7,37	7,41	7,5	7,58	7,72	7,88	8,04	8,14
	**AL/RC**	**Female**	50	2,73	2,78	2,81	2,88	2,93	2,97	2,99	3,01
		**Male**	45	2,8	2,81	2,82	2,88	2,93	2,98	3,02	3,04
		**Total**	95	2,74	2,81	2,82	2,88	2,93	2,98	3,01	3,04
	**ACD**	**Female**	50	3,1	3,14	3,21	3,29	3,58	3,72	3,84	3,96
		**Male**	45	3,08	3,23	3,3	3,39	3,55	3,68	3,76	3,79
		**Total**	95	3,08	3,15	3,23	3,31	3,56	3,7	3,8	3,87
	**PVD**	**Female**	50	2,53	2,62	2,64	2,72	3,01	3,17	3,3	3,4
		**Male**	45	2,53	2,67	2,72	2,86	3,01	3,12	3,19	3,25
		**Total**	95	2,53	2,63	2,69	2,76	3,01	3,13	3,25	3,31

	**LT**	**Female**	50	3,29	3,35	3,37	3,4	3,55	3,67	3,76	3,79
		**Male**	45	3,24	3,29	3,29	3,36	3,47	3,59	3,8	3,9
		**Total**	95	3,24	3,29	3,34	3,39	3,5	3,65	3,76	3,82
	**CCR**	**Female**	50	0,51	0,51	0,52	0,52	0,55	0,57	0,58	0,59
		**Male**	45	0,5	0,51	0,51	0,53	0,55	0,57	0,59	0,59
		**Total**	95	0,5	0,51	0,52	0,52	0,55	0,57	0,58	0,59
**8 years**	**AL**	**Female**	51	21,4	21,46	21,94	22,2	22,45	23,05	23,45	23,62
		**Male**	48	21,9	22,17	22,33	22,7	22,99	23,57	24,03	24,21
		**Total**	99	21,4	21,94	22,12	22,3	22,85	23,34	23,86	24,03
	**CR**	**Female**	51	7,35	7,45	7,55	7,59	7,77	7,88	8,04	8,08
		**Male**	48	7,49	7,5	7,54	7,69	7,82	8,01	8,2	8,29
		**Total**	99	7,36	7,49	7,54	7,64	7,8	7,96	8,11	8,25
	**AL/RC**	**Female**	51	2,8	2,81	2,83	2,85	2,92	2,98	3	3,02
		**Male**	48	2,8	2,85	2,86	2,9	2,94	2,99	3,03	3,07
		**Total**	99	2,8	2,83	2,84	2,87	2,93	2,99	3,02	3,03
	**ACD**	**Female**	51	3,06	3,1	3,16	3,24	3,48	3,68	3,8	3,9
		**Male**	48	3,14	3,2	3,35	3,42	3,61	3,74	3,83	3,96
		**Total**	99	3,09	3,15	3,2	3,35	3,55	3,7	3,81	3,96
	**PVD**	**Female**	51	2,5	2,56	2,64	2,7	2,9	3,1	3,24	3,3
		**Male**	48	2,55	2,64	2,76	2,86	3,06	3,18	3,28	3,37
		**Total**	99	2,51	2,58	2,66	2,79	3	3,13	3,27	3,37
	**LT**	**Female**	51	3,3	3,35	3,37	3,39	3,52	3,68	3,85	3,88
		**Male**	48	3,27	3,28	3,3	3,33	3,47	3,6	3,66	3,74
		**Total**	99	3,28	3,3	3,31	3,38	3,47	3,64	3,81	3,88
	**CCR**	**Female**	51	0,51	0,51	0,52	0,53	0,55	0,57	0,58	0,59
		**Male**	48	0,51	0,52	0,53	0,54	0,56	0,58	0,59	0,61
		**Total**	99	0,51	0,51	0,53	0,53	0,56	0,58	0,59	0,6
**9 years**	**AL**	**Female**	44	21,6	21,84	21,86	22,3	22,88	23,34	23,67	23,82
		**Male**	40	22,2	22,5	22,59	22,8	22,98	23,41	23,94	24,12
		**Total**	84	21,6	22,12	22,25	22,6	22,95	23,35	23,82	23,97
	**CR**	**Female**	44	7,4	7,44	7,48	7,62	7,81	7,92	8,02	8,07
		**Male**	40	7,45	7,5	7,54	7,62	7,78	7,99	8,12	8,16
		**Total**	84	7,41	7,48	7,5	7,62	7,8	7,93	8,05	8,16
	**AL/RC**	**Female**	44	2,82	2,82	2,86	2,88	2,95	2,99	3,02	3,03
		**Male**	40	2,75	2,84	2,88	2,91	2,97	3,02	3,05	3,07
		**Total**	84	2,77	2,82	2,86	2,9	2,96	3	3,03	3,05
	**ACD**	**Female**	44	3,21	3,24	3,29	3,38	3,48	3,66	3,83	3,9
		**Male**	40	3,24	3,29	3,33	3,4	3,56	3,76	3,99	4,07
		**Total**	84	3,21	3,27	3,3	3,39	3,49	3,69	3,9	4,01

	**PVD**	**Female**	44	2,67	2,7	2,72	2,82	2,92	3,06	3,27	3,35
		**Male**	40	2,67	2,73	2,76	2,84	2,98	3,19	3,36	3,48
		**Total**	84	2,67	2,71	2,75	2,82	2,95	3,14	3,33	3,4
	**LT RE**	**Female**	44	3,24	3,27	3,3	3,36	3,47	3,6	3,67	3,7
		**Male**	40	3,2	3,26	3,31	3,37	3,49	3,59	3,68	3,79
		**Total**	84	3,2	3,27	3,3	3,37	3,48	3,59	3,68	3,75
	**CCR**	**Female**	44	0,5	0,5	0,52	0,52	0,55	0,57	0,6	0,61
		**Male**	40	0,5	0,51	0,53	0,54	0,56	0,59	0,63	0,64
		**Total**	84	0,5	0,51	0,52	0,53	0,55	0,58	0,61	0,63
**10 years**	**AL**	**Female**	49	21,8	21,93	22,12	22,4	22,78	23,17	23,54	23,64
		**Male**	41	21,8	22,14	22,5	22,9	23,58	23,93	24,21	24,57
		**Total**	90	21,8	21,96	22,21	22,5	23,06	23,64	23,97	24,21
	**CR**	**Female**	49	7,37	7,43	7,47	7,62	7,73	7,96	8,07	8,13
		**Male**	41	7,7	7,77	7,79	7,84	7,94	8,09	8,28	8,28
		**Total**	90	7,41	7,47	7,62	7,68	7,88	8,03	8,15	8,28
	**AL/RC**	**Female**	49	2,79	2,84	2,85	2,88	2,94	3	3,03	3,05
		**Male**	41	2,76	2,85	2,87	2,89	2,93	3	3,03	3,05
		**Total**	90	2,77	2,84	2,86	2,89	2,94	3	3,03	3,05
	**ACD**	**Female**	49	3	3,11	3,24	3,36	3,53	3,67	3,87	3,9
		**Male**	41	3,21	3,25	3,28	3,41	3,65	3,84	3,94	4,01
		**Total**	90	3,09	3,22	3,27	3,37	3,56	3,73	3,89	3,98
	**PVD**	**Female**	49	2,45	2,6	2,7	2,83	2,97	3,11	3,34	3,36
		**Male**	41	2,69	2,73	2,75	2,84	3,08	3,24	3,4	3,48
		**Total**	90	2,54	2,66	2,72	2,84	2,99	3,2	3,35	3,46
	**LT**	**Female**	49	3,26	3,27	3,33	3,41	3,48	3,54	3,8	3,82
		**Male**	41	3,2	3,22	3,28	3,31	3,46	3,51	3,6	3,71
		**Total**	90	3,2	3,27	3,28	3,34	3,47	3,54	3,7	3,82
	**CCR**	**Female**	49	0,5	0,5	0,52	0,53	0,55	0,57	0,59	0,59
		**Male**	41	0,5	0,51	0,52	0,53	0,56	0,58	0,6	0,61
		**Total**	90	0,5	0,51	0,52	0,53	0,55	0,58	0,59	0,61
**11 years**	**AL**	**Female**	58	21,9	22,03	22,14	22,5	22,87	23,28	23,65	23,84
		**Male**	39	22,2	22,59	22,65	23,1	23,63	23,99	24,42	24,6
		**Total**	97	21,9	22,14	22,31	22,6	23,09	23,65	24,07	24,42
	**CR**	**Female**	58	7,33	7,48	7,52	7,6	7,74	7,84	7,98	8,12
		**Male**	39	7,44	7,54	7,6	7,69	7,87	8	8,11	8,18
		**Total**	97	7,35	7,51	7,54	7,65	7,81	7,91	8,04	8,15
	**AL/RC**	**Female**	58	2,84	2,88	2,89	2,92	2,96	3	3,05	3,08
		**Male**	39	2,87	2,92	2,92	2,95	3	3,05	3,1	3,15
		**Total**	97	2,87	2,88	2,91	2,93	2,97	3,01	3,08	3,12
	**ACD**	**Female**	58	3,1	3,27	3,3	3,37	3,54	3,65	3,76	3,95

		**Male**	39	3,22	3,37	3,47	3,59	3,78	3,93	4,08	4,17
		**Total**	97	3,16	3,29	3,36	3,44	3,63	3,78	3,96	4,07
	**PVD**	**Female**	58	2,57	2,69	2,74	2,84	3	3,1	3,23	3,38
		**Male**	39	2,67	2,82	2,9	3,02	3,2	3,34	3,51	3,6
		**Total**	97	2,6	2,73	2,79	2,9	3,07	3,22	3,41	3,48
	**LT**	**Female**	58	3,29	3,32	3,34	3,41	3,5	3,57	3,7	3,72
		**Male**	39	3,11	3,19	3,2	3,29	3,4	3,51	3,7	3,95
		**Total**	97	3,18	3,25	3,3	3,35	3,47	3,57	3,7	3,72
	**CCR**	**Female**	58	0,51	0,52	0,52	0,53	0,55	0,56	0,58	0,59
		**Male**	39	0,5	0,52	0,53	0,54	0,56	0,59	0,62	0,62
		**Total**	97	0,51	0,52	0,52	0,53	0,55	0,57	0,59	0,62
**Total**	**AL**	**Female**	299	21,4	21,61	21,86	22,1	22,59	23,12	23,5	23,65
		**Male**	259	21,8	22,01	22,29	22,6	23,04	23,64	24,07	24,27
		**Total**	558	21,5	21,81	22	22,3	22,86	23,34	23,84	24,07
	**CR**	**Female**	299	7,35	7,41	7,47	7,56	7,74	7,87	8,03	8,09
		**Male**	259	7,48	7,53	7,57	7,68	7,83	8,02	8,16	8,28
		**Total**	558	7,38	7,47	7,52	7,61	7,79	7,94	8,1	8,21
	**AL/RC**	**Female**	299	2,79	2,82	2,85	2,88	2,93	2,98	3,02	3,04
		**Male**	259	2,78	2,83	2,86	2,9	2,94	3	3,05	3,07
		**Total**	558	2,79	2,83	2,85	2,88	2,93	2,99	3,03	3,06
	**ACD**	**Female**	299	3,09	3,16	3,23	3,31	3,51	3,65	3,81	3,9
		**Male**	259	3,15	3,23	3,31	3,42	3,59	3,75	3,93	4,02
		**Total**	558	3,11	3,2	3,26	3,36	3,55	3,7	3,86	3,96
	**PVD**	**Female**	299	2,53	2,62	2,69	2,77	2,95	3,1	3,26	3,35
		**Male**	259	2,57	2,69	2,75	2,86	3,04	3,19	3,35	3,46
		**Total**	558	2,55	2,64	2,71	2,81	2,99	3,15	3,32	3,4
	**LT**	**Female**	299	3,27	3,32	3,36	3,4	3,52	3,63	3,74	3,84
		**Male**	259	3,2	3,26	3,29	3,33	3,47	3,6	3,72	3,83
		**Total**	558	3,24	3,29	3,32	3,38	3,5	3,63	3,72	3,83
	**CCR**	**Female**	299	0,5	0,51	0,52	0,53	0,55	0,57	0,58	0,59
		**Male**	259	0,5	0,51	0,52	0,54	0,56	0,58	0,6	0,62
		**Total**	558	0,5	0,51	0,52	0,53	0,55	0,57	0,59	0,61

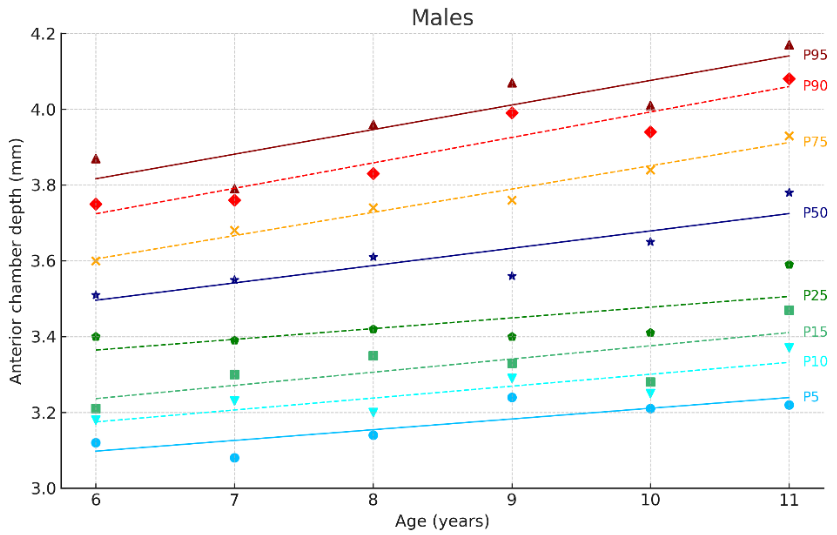

Figure S1. ACD growth curves of the eye for Males aged 6 to 11 Years. 5 to 95 represent the 5th to 95th percentiles. Males N = 259 (6 years = 45, 7 years = 45, 8 years = 48, 9 years = 40, 10 years = 41, 11 years = 39).

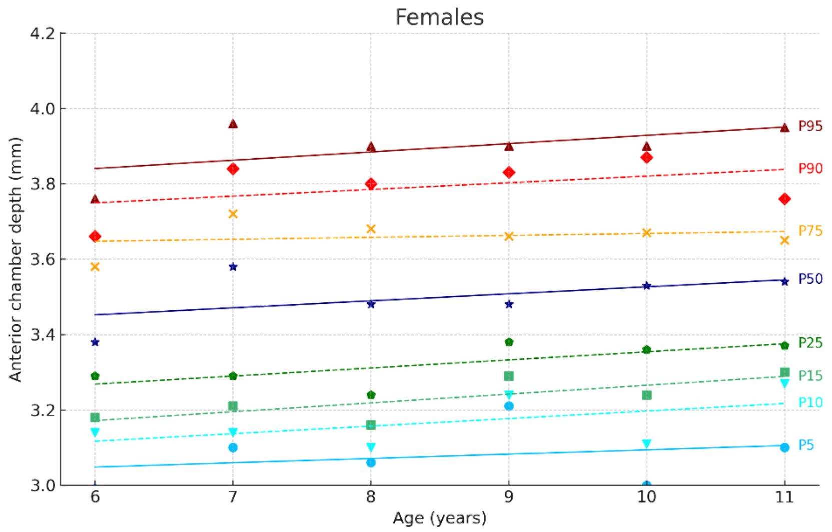

Figure S2. ACD growth curves of the eye for Females aged 6 to 11 Years. 5 to 95 represent the 5th to 95th percentiles. Females N = 299 (6 years = 47, 7 years = 50, 8 years = 51, 9 years = 44, 10 years = 49, 11 years = 58).

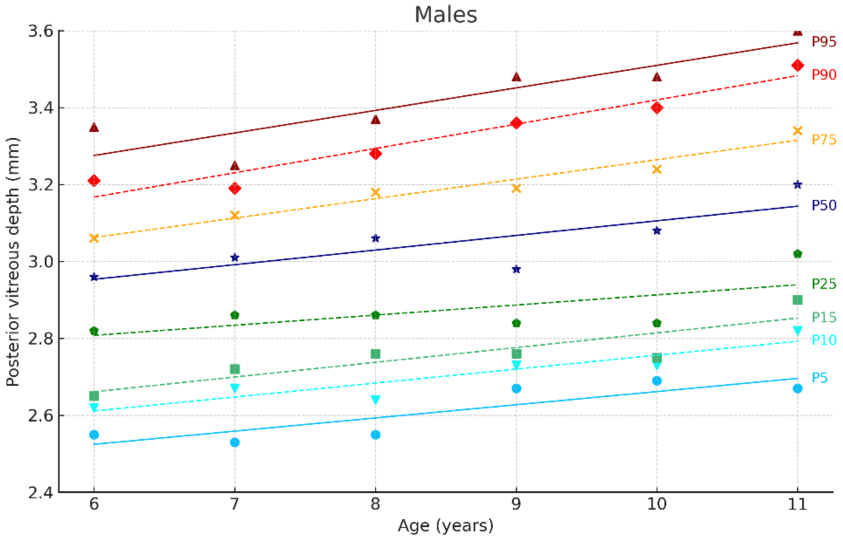

Figure S3. PVD growth curves of the eye for Males aged 6 to 11 Years. 5 to 95 represent the 5th to 95th percentiles. Males N = 259 (6 years = 45, 7 years = 45, 8 years = 48, 9 years = 40, 10 years = 41, 11 years = 39).

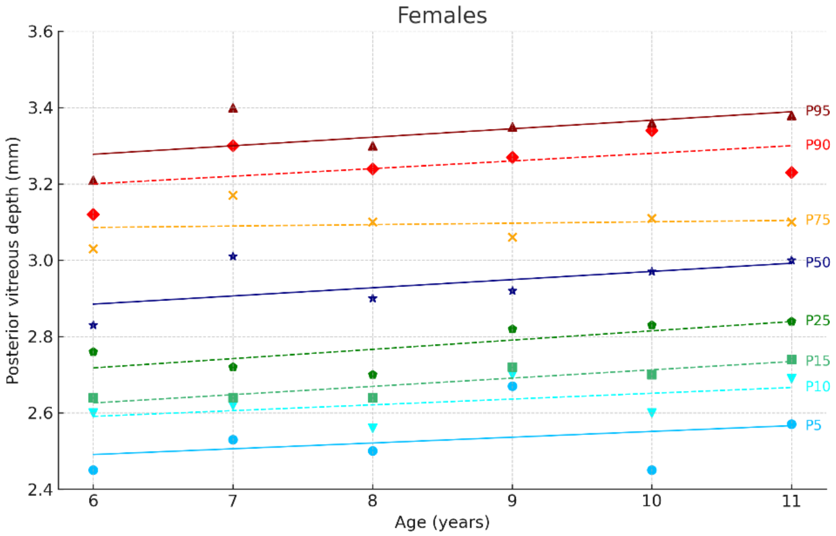

Figure S4. PVD growth curves of the eye for Females aged 6 to 11 Years. 5 to 95 represent the 5th to 95th percentiles. Females N = 299 (6 years = 47, 7 years = 50, 8 years = 51, 9 years = 44, 10 years = 49, 11 years = 58).

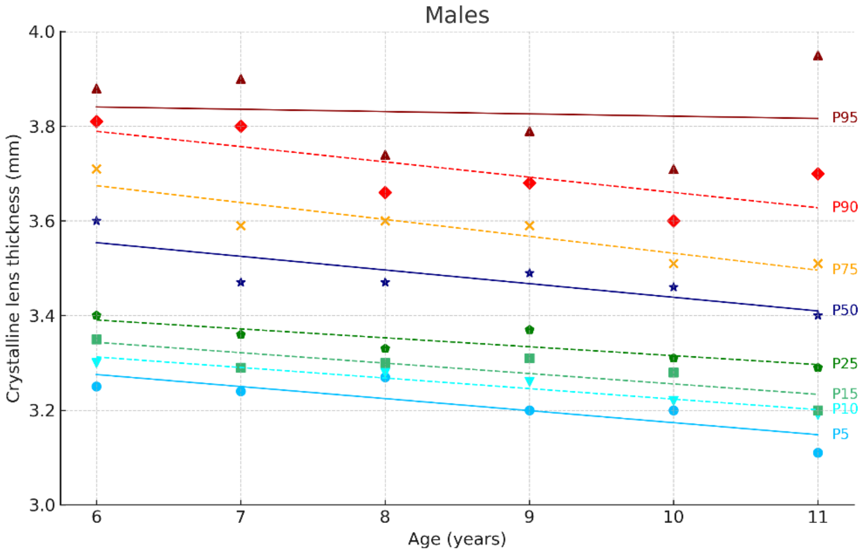

Figure S5. LT growth curves of the eye for Males aged 6 to 11 Years. 5 to 95 represent the 5th to 95th percentiles. Males N = 259 (6 years = 45, 7 years = 45, 8 years = 48, 9 years = 40, 10 years = 41, 11 years = 39).

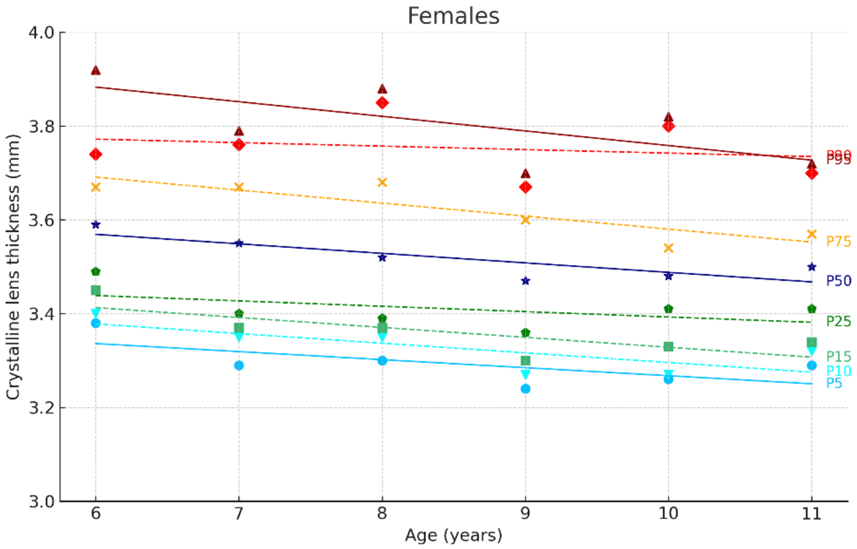

Figure S6. LT growth curves of the eye for Females aged 6 to 11 Years. 5 to 95 represent the 5th to 95th percentiles. Females N = 299 (6 years = 47, 7 years = 50, 8 years = 51, 9 years = 44, 10 years = 49, 11 years = 58).

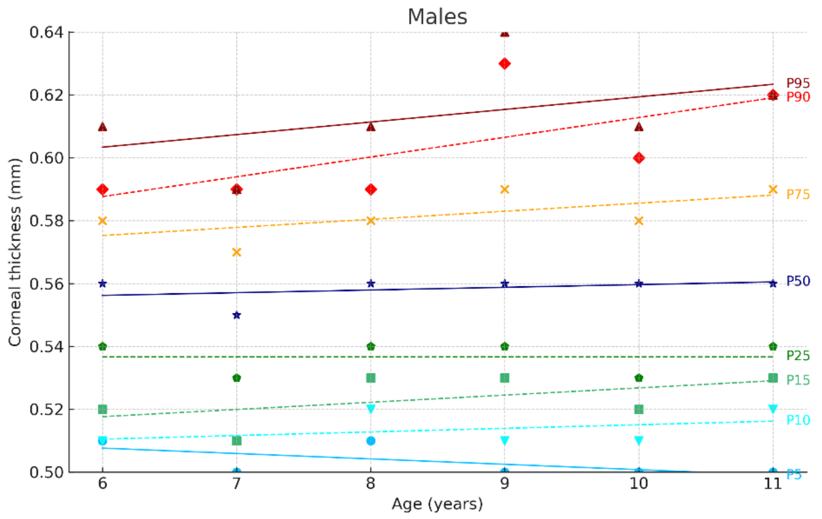

Figure S7. CCT growth curves of the eye for Males aged 6 to 11 Years. 5 to 95 represent the 5th to 95th percentiles. Males N = 259 (6 years = 45, 7 years = 45, 8 years = 48, 9 years = 40, 10 years = 41, 11 years = 39).

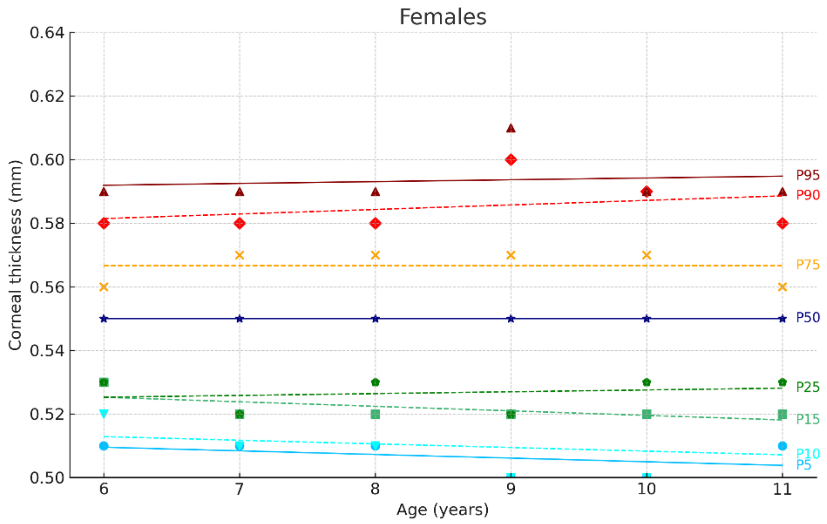

Figure S8. CCT growth curves of the eye for Males and Females aged 6 to 11 Years. 5 to 95 represent the 5th to 95th percentiles. Females N = 299 (6 years = 47, 7 years = 50, 8 years = 51, 9 years = 44, 10 years = 49, 11 years = 58).

Table S2. (a) 6 year: Comparison this study vs. Tideman, 2018 [17]. Sampling simulation. (b) 9year: Comparison this study vs. Tideman, 2018 [17]. Sampling simulation.

(a)

**6 years**						**CL 95%**			
				**Bias**	**Standard error**	**Lower**	**Upper**	**Media Tideman, 2018**	**Significance**
**AL**	Female	5	20,9652	0,0009	0,34011	20,32	21,448049	20,96	NO
		10	21,4294	-0,093116	0,19667	20,96	21,541126	21,22	NO
		25	21,7	-0,03668	0,1228	21,453	21,9	21,66	NO
		50	22,054	-0,002489	0,1236	21,846	22,304	22,06	NO
		75	22,638	-0,024223	0,1816	22,27325	22,95925	22,49	NO
		90	23,1642	0,00334	0,23169	22,769	23,772	22,86	NO
		95	23,6144	-0,075582	0,26882	23,030713	23,888	23,11	NO
	Male	5	21,774	-0,082851	0,22317	21,216	21,961531	21,42	NO
		10	21,8816	0,013961	0,1142	21,768	22,202441	21,71	SI
		25	22,3605	-0,057885	0,17581	21,984	22,59125	22,14	NO
		50	22,827	-0,028965	0,11059	22,557	23,018073	22,59	NO
		75	23,224	-0,023086	0,13607	22,931	23,406456	23,01	NO
		90	23,4468	0,036032	0,14208	23,291691	23,872	23,41	NO
		95	23,7712	-0,029905	0,25948	23,40561	24,19	23,65	NO
**CR**	Female	5	7,2771	-0,0379	0,1056	7,0009	7,4012	7,32	NO
		10	7,3016	0,0382	0,0643	7,267	7,4614	7,41	NO
		25	7,4901	-0,0018	0,0358	7,4059	7,5545	7,54	NO
		50	7,6277	0,0053	0,0531	7,5523	7,7378	7,7	NO
		75	7,8005	-0,0014	0,0343	7,7346	7,8739	7,85	NO
		90	8,0276	-0,029	0,0974	7,826	8,1498	8	NO
		95	8,1266	0,0036	0,0924	7,9194	8,3008	8,11	NO
	Male	5	7,456	0,0052	0,0542	7,3711	7,5573	7,42	NO
		10	7,5184	0,0093	0,045	7,4378	7,6288	7,52	NO
		25	7,6895	-0,0125	0,0535	7,5645	7,7676	7,68	NO
		50	7,8157	0,0181	0,0538	7,7566	7,9367	7,84	NO
		75	8,0928	-0,0259	0,0764	7,9235	8,193	8	NO
		90	8,2181	0,0133	0,0673	8,1166	8,3674	8,16	NO
		95	8,3531	0,0171	0,1316	8,193	8,6282	8,27	NO
**AL/RC**	Female	5	2,7754	-0,0026	0,0214	2,7254	2,8159	2,75	NO
		10	2,7899	0,0087	0,0197	2,7739	2,8422	2,78	NO
		25	2,8498	0,0005	0,0144	2,8189	2,8744	2,82	NO
		50	2,8942	-0,0016	0,0108	2,8744	2,9211	2,87	NO
		75	2,9377	0,0049	0,0175	2,913	2,9806	2,91	NO
		90	2,9914	0,0024	0,0197	2,9501	3,0488	2,95	NO
		95	3,0356	-0,0104	0,0238	2,9823	3,0516	2,97	SI

	5	2,7581	-0,007	0,0489	2,6551	2,8232	2,75	NO
	10	2,8002	0,0013	0,0242	2,7484	2,8317	2,79	NO
	25	2,8494	-0,0017	0,0154	2,8246	2,8738	2,84	NO
Male	50	2,9012	-0,0035	0,0153	2,8696	2,9222	2,89	NO
	75	2,9379	0,0018	0,0121	2,9185	2,9585	2,92	NO
	90	2,9662	0,0069	0,0222	2,9523	3,0343	2,96	NO
	95	3,0215	-0,0083	0,033	2,9593	3,0607	2,99	NO

a. Unless otherwise stated, sampling simulation results are based on 1 000 sampling simulation samples.

(b)

**9 years**		**Sex**	**Percentil**	**Percentil**	**Bias**	**Standard error**	**95% CL** Lower	Upper	**Tideman, 2018**	**Significance**
	**ALOD**	Female	5	21,366	0,000057	0,299655	20,834	21,852855	21,62	NO
			10	21,725	0,012134	0,217065	21,289	22,1975	21,9	NO
			25	22,2655	0,014174	0,177243	21,85525	22,626	22,33	NO
			50	22,879	0,031506	0,155156	22,578	23,1645	22,79	NO
			75	23,34725	0,021134	0,102239	23,157	23,626771	23,25	NO
)			90	23,68	0,024965	0,165975	23,405142	23,966	23,73	NO
			95	23,92925	0,12982	0,369702	23,625246	24,734	24,04	NO
		Male	5	22,2225	-0,324067	0,621379	20,766	22,57	22,09	NO
			10	22,4511	-0,035122	0,256522	21,770122	22,693873	22,39	NO
			25	22,75375	-0,005025	0,098935	22,57	22,91125	22,83	NO
			50	22,9765	0,009943	0,084787	22,8585	23,21	23,31	NO
			75	23,43575	0,007533	0,195017	23,101	23,852316	23,79	NO
			90	23,9447	-0,008092	0,167894	23,538	24,236187	24,28	SI
			95	24,2162	-0,071355	0,153345	23,825573	24,321	24,6	SI
	**CROD**	Female	5	7,3742	-0,0128	0,0617	7,2454	7,47	7,34	NO
			10	7,4223	0,0113	0,0464	7,3649	7,5181	7,42	NO
			25	7,603	0,016	0,0969	7,4756	7,7794	7,56	SI
			50	7,8141	0,0033	0,032	7,7656	7,8888	7,72	SI

		75	7,9202	0,0006	0,0238	7,8653	7,9637	7,88	NO
		90	8,0324	0,0241	0,1146	7,9275	8,3622	8,02	NO
		95	8,2901	-0,0307	0,1694	7,9968	8,4805	8,13	NO
	Male	5	7,4246	0,0243	0,0392	7,4054	7,5249	7,43	NO
		10	7,497	0,0032	0,0411	7,4198	7,5794	7,53	NO
		25	7,612	0,0083	0,0601	7,526	7,7421	7,69	NO
		50	7,7821	0,0136	0,053	7,7238	7,8967	7,84	NO
		75	7,9867	-0,0102	0,0461	7,8828	8,054	8,02	NO
		90	8,1332	-0,0186	0,0626	7,9964	8,2124	8,17	NO
		95	8,1631	0,049	0,0999	8,054	8,3878	8,27	NO
**RatioLA CR RE**	Fem ale	5	2,7836	0,0066	0,0294	2,7468	2,852	2,82	NO
		10	2,8193	0,0094	0,0261	2,7726	2,8704	2,86	NO
		25	2,878	0,0014	0,0126	2,8609	2,9166	2,91	NO
		50	2,9455	-0,0013	0,0157	2,9006	2,9683	2,95	NO
		75	2,9879	-0,0009	0,0138	2,9649	3,0109	3	NO
		90	3,0153	0,0024	0,0104	2,9987	3,0403	3,05	SI
		95	3,0372	-0,0017	0,0152	3,0121	3,0601	3,09	SI
	Male	5	2,7453	0,0072	0,048	2,6944	2,8616	2,84	NO
		10	2,8216	-0,0018	0,0561	2,7346	2,9083	2,87	NO
		25	2,9108	0,0058	0,0193	2,8617	2,9507	2,92	NO
		50	2,9698	-0,002	0,0132	2,944	2,9956	2,97	NO
		75	3,0191	-0,0038	0,0145	2,9805	3,0361	3,02	NO
		90	3,0516	0,0017	0,0209	3,0235	3,1015	3,07	NO
		95	3,0878	0,001	0,0361	3,0361	3,1504	3,12	NO

a Unless otherwise stated, sampling simulation results are based on 1 000 sampling simulation samples
